# Effects of Indoor Air Pollutants on Atopic Dermatitis

**DOI:** 10.3390/ijerph13121220

**Published:** 2016-12-09

**Authors:** JaKyoung Kim, HyungJin Kim, DaeHyun Lim, Young-Kyu Lee, Jeong Hee Kim

**Affiliations:** 1 Department of Pediatrics, School of Medicine, Kangwon National University, 1 Kangwondaehak-gil, Chuncheon-si, Gangwon-do 24341, Korea; kjaky@kangwon.ac.kr; 2 Department of Pediatrics, Inha University School of Medicine, 100 Inha-ro, Nam-gu, Incheon 22212, Korea; jy8318@nate.com (H.K.); dhnlim@naver.com (D.L.); 3 Environmental Health Center for Allergic Rhinitis, Inha University Hospital, 27 Inha-ro, Jung-gu, Incheon 22332, Korea; 4 Indoor Air Quality Analysis Center, National Instrumentation Center for Environmental Management, Seoul National University, Seoul 08826, Korea; woodlee9@snu.ac.kr

**Keywords:** dermatitis, atopic, air pollution, indoor, VOCs, formaldehyde

## Abstract

The increasing prevalence of atopic dermatitis (AD) is associated with variations in indoor environments. In Korea, many inner walls of homes are covered with wallpaper: such walls emit indoor air pollutants, including volatile organic compounds (VOCs) and formaldehyde. This randomized, double-blind study investigated the effects of wallpaper on indoor air quality and AD. Thirty-one children (aged three to eight years) with moderate AD were assigned to environmentally-friendly (EF) and polyvinyl chloride (PVC) wallpaper groups. Indoor air concentrations of VOCs, natural VOCs (NVOCs), formaldehyde, and total suspended bacteria were measured before and two (W_2_) and eight weeks (W_8_) after wallpapering. Scoring Atopic Dermatitis (SCORAD) evaluations and blood tests were performed during the same period. The EF wallpaper and PVC wallpaper groups showed similar trends in the changes in total VOCs (TVOC) and formaldehyde content in the indoor air. However, the EF wallpaper group showed more improvement on the SCORAD at W_2_ and W_8_ than the PVC wallpaper group. The SCORAD index was positively correlated with several indoor air pollutants. Further, the SCORAD index and NVOC % were negatively correlated. Improved SCORAD index and effects of wallpapering on indoor air quality improvements occurred within a short period of time in both groups. We believe that NVOCs in indoor air after EF wallpapering have a beneficial effect on health.

## 1. Introduction

The increasing prevalence of allergic diseases, particularly asthma, in Korea is associated with a higher number of people living in apartment buildings and a higher number of cars since the mid-1980s [1]. A recent study demonstrated that the prevalence of atopic dermatitis (AD) symptoms in children aged six to seven years in Korea was 5.7% and 11.2% in 2000 and 2010, respectively [2]. There is a considerable body of evidence showing the relationship between outdoor air pollutants and allergic diseases. However, the indoor environment is of particular interest with regard to its role in the development and aggravation of allergic diseases because young children spend most of their time indoors [3,4]. Many studies have reported the relationship between indoor air pollutants and asthma [5,6], but fewer have reported the effects of indoor air pollutants on AD, a common and troublesome condition among young children [7,8,9].

Formaldehyde, volatile organic compounds (VOCs), and aromatic compounds are major indoor environmental pollutants. They are emitted by new furniture and finishing materials such as wallpaper, plywood, and polyvinyl chloride (PVC) flooring [4]. A previous study found that the main source of indoor VOCs and formaldehyde in Korean houses was wallpaper, rather than new furniture or flooring, because wallpaper covers the inner walls and ceilings of houses and has a surface area that is three to four times greater than that of the floor [10]. According to published literature, indoor air pollutants may play a key role in the development and aggravation of allergic diseases [4]. To improve indoor air quality, many products have been made less toxic; as an example, environmentally friendly (EF) wallpaper, without PVCs, is now available. The purpose of this study was to evaluate the effects of wallpapering on indoor air quality and symptoms of AD in children. To the best of our knowledge, this is the first study to measure indoor air pollutant concentrations after EF wallpapering compared to PVC wallpapering. In addition, the Scoring Atopic dermatitis (SCORAD) index in children was calculated before and after wallpapering. The correlation between the concentrations of pollutants and symptoms of AD was evaluated.

## 2. Materials and Methods

### 2.1. Study Participants

Study participants, aged three to eight years, all residing in apartment houses, were recruited via wall posters from January to April 2010 at Inha University and Kangwon National University Hospital. They were examined for AD according to the criteria reported by Hanifin and Rajka [11]. The SCORAD index was used to evaluate the severity of AD; AD was classified as mild (<25 points), moderate (25–50 points), and severe (>50 points) [12]. Children who scored 25–50 points were enrolled. Informed consent was obtained from all of the participants’ parents. The study was approved by the Institutional Review Board (IRB) at both hospitals (Inha IRB protocol number: IUH-IRB 10-0311 and Kangwon IRB protocol number: 09-30). A questionnaire was administered to assess comorbidities and family history of allergic diseases (AD, asthma, and allergic rhinitis), type of housing, and indoor renovations in the past year. Allergen testing was performed using a skin prick test with 38 allergens (Allergopharma, Reinbek, Germany). The allergens included house dust mite allergens (*Dermatophagoides pteronyssinus* and *D. farina*), animal fur (cat and dog), fungi (*Aspergillus*, *Alternaria*, *Candida* spp., etc.), pollen (from alder, oak, birch, ryegrass, mugwort, ragweed, and Japanese hop) and food allergens (milk, egg white, soy bean, wheat, shrimp, peanut, etc.). If the wheal size for an allergen was larger than that for the positive control (i.e., 1% histamine), the skin test was considered positive.

**Figure 1:**
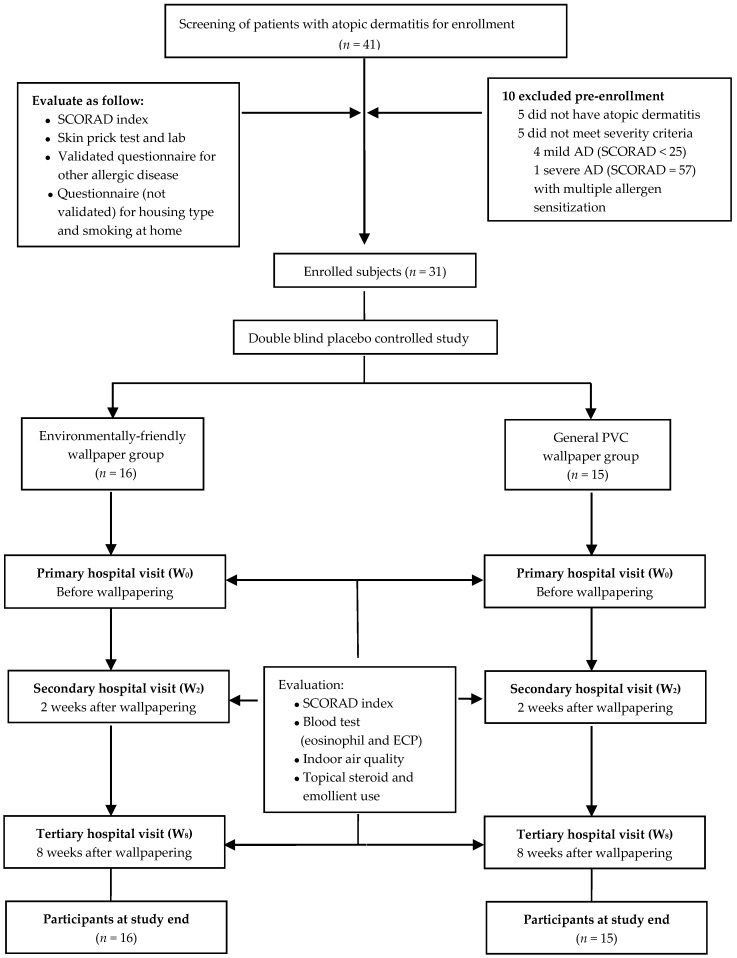
Study participants and design. AD: Atopic dermatitis; SCORAD: Scoring Atopic Dermatitis; PVC: Polyvinyl chloride; ECP: Eosinophil cationic protein.

The exclusion criteria included severe AD (>50). In addition, children with food allergies and who showed sensitization to multiple antigens, except house dust mites, were excluded. Children who spent <12 h indoors daily or who lived in a house that was renovated or newly built within the past year were also excluded. Furthermore, children who lived in detached houses were excluded because the ventilation systems in such houses differ from those in apartments. No included study participants lived in a house that was constructed or renovated within the past two years. All participants’ families were discouraged from buying new furniture or renovating during the study period to minimize any environmental changes, except those associated with wallpapering. A total of 41 children were recruited, but 10 were excluded from this study for the following reasons: five children did not have AD, and the other five children did not meet the severity criteria of AD. Therefore, 31 participants took part in this study (Figure 1).

#### Questionnaires

Study participants and parents were asked to fill out two questionnaires. One questionnaire was about allergic comorbidities and was based on a Korean version of the International Study of Asthma and Allergies in Childhood (ISAAC) which has 31 questions [1,13]. The other was a questionnaire, which has not been validated, and included the family history of allergies, type of housing, the frequency of ventilation over a day, the frequency of cleansing over a day, the smoking status at home, and the use of air freshener before wallpapering. After wallpapering, we investigated the frequency of ventilation over a day, the smoking status at home, and the frequency and volume of topical steroid use using this questionnaire.

### 2.2. Study Design

The study design is shown in Figure 1. Participants were randomly divided into two groups (the EF-wallpaper and PVC-wallpaper groups) and a double-blind design was used. The living room and participant’s room, where the participant spent most of his/her time, were selected for wallpapering. The old wallpaper was removed before the new one was installed. We used two kinds of wallpaper: EF wallpaper, which is manufactured using plant material such as kenaf, and PVC-coated wallpaper [14]. The PVC wallpaper looked similar to the EF wallpaper regarding color and texture selection. The same natural adhesives were used for wallpapering either type. The natural adhesives used were wheat-based and commonly used for wallpapering in Korea. Typically, such adhesives had a 1:2 water:wheat ratio and are made using a heating-and-mixing method that does not include VOCs and formaldehyde.

Before wallpapering (W_0_), and two (W_2_) and eight weeks (W_8_) after wallpapering, the quality of the indoor air was evaluated. In addition, the participants’ SCORAD index, serum eosinophil count, and eosinophil cationic protein (ECP) levels were measured to assess improvement in AD. The amount and times moisturizer (Zeroid^®^, Daejeon, Korea, ceramide-containing lotion) and topical steroids (prednicarbate 0.25% cream) were applied were recorded. Participants’ parents were directed to apply moisturizer twice a day on the participants and the topical steroid was to be used as needed to heal itching. In addition, they were instructed not to provide the participants with any medicines, such as antihistamines, until the end of the study. If participants’ symptoms were sufficiently aggravated to demand any other treatment, they were taken off the study, thus excluding these participants from the study cohort.

### 2.3. Inflammatory Markers for AD

The eosinophil count, total immunoglobulin E (IgE), and ECP levels were considered to be the inflammatory markers of AD. The eosinophil count in the peripheral blood was measured using an automated hematology analyzer, Sysmex XE-2100 (Sysmex, Kobe, Japan). Total IgE level in the serum was analyzed using a total IgE kit (IgE FEIA/TEMPO, Pharmacia AB, Uppsala, Sweden). ECP analysis was performed using fluoroimmunoassays, according to the manufacturer’s instructions (Pharmacia AB).

### 2.4. Indoor Air Pollutant Measurements

The study was conducted between May and October 2010. We chose this timing because the heat is not turned on during this period in houses in Korea. The measurements were performed at W_0_, W_2_, and W_8_ according to the process established by the Indoor Air Quality (IAQ) Act of Korea [15]. All sampling was conducted during the daytime to ensure similar environmental conditions. Samples were collected from two locations—the living room and participant’s bedroom—where the wallpaper was changed. Furthermore, samples were collected after closing the rooms for an hour, which was preceded by 20 min of natural ventilation. The temperature and relative humidity were not controlled.

#### 2.4.1. Measurement of VOCs, Natural VOCs (NVOCs), and Formaldehyde

The measurement equipment was installed at a height of 1.2–1.5 m from the floor and >1 m from the wall to collect samples using a portable pump (∑30, SIBATA, Japan for VOCs and ∑100, SIBATA for formaldehyde). VOCs were sampled via use of a Tenax TA tube (Supelco, Bellefonte, PA, USA), and formaldehyde was collected with a 2,4-dinitrophenyhydrazine (2,4-DNPH) cartridge. Data were collected for 30 min respectively at air volumes of 3.2 and 30 L, wrapped in a sample tube, and then stored at 4 °C until further analysis.

The compounds absorbed by the Tenax TA were analyzed by a thermal desorption system (TDS, Gerstel, Műlheim, Germany) and gas chromatography (7890N, Agilent, Santa Clara, CA, USA)/mass spectrometry system (5975, Agilent, Santa Clara, CA, USA). Calibration and quantification of all samples were performed according to their respective standards (52 Component Indoor Air Standards, Supelco) under the same conditions as those presented in Table 1 and Table 2. Benzene, toluene, ethylbenzene, xylene, styrene, and 2-ethyl-1-hexanol (2E1H) levels, which are named 5VOCs, were measured. The concentration of total VOCs (TVOC) was estimated by the sum of the peaks from *n*-hexane to *n*-hexadecane, converted using the toluene calibration factor. The method for measuring VOCs was applied to measure the concentrations of NVOCs. NVOCs are emitted from forests and known as phytoncides [16]. Among monoterpenes with *n* = 2, 20 species, including α-pinene, β-pinene, and camphene, were investigated.

Formaldehyde was measured using samples collected with the 2,4-DNPH cartridge. The concentration of formaldehyde was measured using high-performance liquid chromatography (HPLC, Hewlett Packard Series 1100, Golden, CO, USA) under the conditions presented in Table 3.

#### 2.4.2. Measurements of the Concentration of Total Suspended Bacteria (TSB)

Bacterial aerosols were collected by impaction (MAS100-Ecoinstrument, Merck, Stäfa, Switzerland), according to the process outlined in the IAQ Act of Korea [15]. The sampler was located at a height of 1.5 m from the floor and samples were collected once at a flow rate of 100 L/min. Tryptic soy agar was used for culture at 35 °C for 48 h. Bacterial colonies were counted at 24 and 48 h. Finally, we calculated the colony forming units (CFU)/area by dividing the number of colonies with the collected indoor air volume (m^3^).

### 2.5. Sample Size and Statistical Analysis

SAS Proc Power (Version 9.4, SAS Institute Inc., Cary, NC, USA) was used for statistical analysis calculating the sample size and power [17]. The effective rate of formaldehyde and TVOCs was the determining factor for sample size calculation. Assuming the estimated effective rate in the EF and PVC-wallpaper groups, respectively, 12 patients in each group are necessary to detect a statistically significant difference between the two groups using a two-sided test at 80% power and α = 0.05.

Data were analyzed using the generalized linear model procedure (PROC GLIM in SAS v9.3) to fit a mixed mode. Each measurement was repeated three times for each participant, and the individual identification number was included as a random effect. The model was adjusted for the effects of gender, age, and amount of emollient used. A two-sample *t*-test and an exact Wilcoxon test were used to compare indoor air quality levels before and after wallpapering between the groups. Indoor air pollutants were transformed to a logarithmic scale as they followed a log-normal distribution. *p* < 0.05 was considered to be statistically significant. We used the beta value to evaluate the relationship between indoor air pollutants and the SCORAD index. The beta value was defined by the SCORAD index/In(μg/m^3^) of the air pollutants.

## 3. Results

### 3.1. Characteristics of Subjects

The study included 31 children. The mean age was 5.4 years, and the male to female ratio was 1:1.17. The EF-wallpaper group consisted of 15 children and the PVC-wallpaper group consisted of 16. There was no significant difference in age and amount of emollient used between the groups (*p* = 0.57 and 0.49, respectively) (Table 4). In addition, the eosinophil count, IgE level, and ECP level were not significantly different between the groups. The SCORAD index before wallpapering was 40.20 ± 11.63 in the EF-wallpaper group and 35.08 ± 7.29 in the PVC-wallpaper group (*p* = 0.10). There was no significant difference in SCORAD index scores between the groups. The objective SCORAD index was defined as the extent of the participant’s inflammatory lesions and intensity, and the subjective score was subtracted from the total score of the SCORAD index.

### 3.2. Indoor Air Quality

The concentration of formaldehyde increased by W_2_ in both groups but decreased by W_8_. The concentration of TVOCs and 5VOCs decreased in both groups by W_2_ and W_8_ (Table 5). Interestingly, the decrease in concentration of TVOCs at W_2_ in the PVC-wallpaper group was significantly higher than that in the EF-wallpaper group. The concentration of NVOC increased by W_2_ but decreased by W_8_ in the EF-wallpaper group; however, in the PVC-wallpaper group, the concentration decreased by W_2_ and W_8_. TSB increased at W_2_ compared to W_0_ and then decreased at W_8_ in both groups.

### 3.3. SCORAD Index and Blood Test Findings

In the EF-wallpaper group, the SCORAD index continued to decrease significantly after wallpapering (*p* = 0.02 by W_2_ and 0.00 by W_8_). In the PVC-wallpaper group, the SCORAD index continued to decrease as well. However, a statistically significant difference in the PVC-wallpaper group was noted only by W_8_ (*p* = 0.00). The objective SCORAD index also continued to decrease after wallpapering in the EF group (*p* = 0.02 by W_2_ and 0.00 by W_8_). However, the objective SCORAD index did not change between W_0_ and W_2_ (*p* = 0.40), and a statistically significant difference was noted only by W_8_ in the PVC-wallpaper group (*p* = 0.00; Table 6). The eosinophil count and ECP levels did not show any statistically significant changes in either groups.

### 3.4. Relationship between Indoor Air Quality and SCORAD Index

We used the beta value to evaluate the relationship between indoor air quality and the SCORAD index. TVOCs and 5VOCs, except styrene, and formaldehyde showed a significant and positive correlation with the SCORAD index. However, NVOC % (i.e., the percentage of NVOCs in TVOCs) showed a significant negative correlation with the SCORAD index (Table 7).

## 4. Discussion

The purpose of our study was to determine the effect of EF wallpaper on indoor air quality and to investigate the association between indoor air quality and symptoms of AD in children. 

The mean concentration of TVOCs from the subjects’ house at W_0_ (1095 ± 883.7, 1099.1 ± 1636.6 in the EF and PVC-wallpaper groups, respectively) exceeded the current Korean threshold for childcare centers (400 μg/m^3^) [15], and the mean concentration of formaldehyde at W_0_ in the EF-wallpaper group was also higher than the recommended threshold (106.3 ± 32.8 and 79.1 ± 22.9 in the EF and PVC-wallpaper groups, respectively). The Korean threshold for formaldehyde in indoor air as specified for an 8 h value is 100 μg/m^3^, which is higher than the recommended threshold of other countries [18]. A previous study reported that the concentration of VOCs in the indoor air of houses of patients with AD and allergic asthma was approximately 1.4–2.0 fold higher than in the houses of the same age group without atopy [7]. In our study, the concentrations of TVOCs were found to be higher than values in previous studies on eco-friendly materials or in the houses of patients with AD [19,20,21]. These high concentrations were similar to the results shown in a previous study that measured the indoor air quality in an unoccupied newly built building [22]. The reason that high levels of TVOCs were measured in our study is due to the use of artificial air fresheners or air cleansing, air ventilation, location, and floor level of the apartment, and the surrounding region/area [21,22]. High concentrations of VOCs or formaldehyde are associated with the onset of Sick Building Syndrome (SBS) or the aggravation of allergic diseases in newly built buildings [4,23,24]. Some studies have reported that exposure to VOCs can damage the epidermal barrier and exacerbate the adverse effects of house dust mites in patients with AD [25,26]. We excluded patients who were sensitized to food or multiple allergens to minimize confounding variables, and AD severity in this study was moderate, not mild. Although we could not evaluate the relationship between AD severity and the concentration of TVOCs, the results suggested that a high concentration of TVOCs had adverse effects on children with AD. We planned wallpapering to improve indoor air quality in terms of indoor air pollutant concentration, and intended to observe changes in indoor air qualities. We expected to see better effects of eco-friendly materials on indoor air quality than the existing materials, such as PVC wallpaper. To sum up, after wallpapering, there was a change in indoor air quality and the concentration of TVOCs continuously decreased by W_2_ and W_8_ in both groups. The concentration of 5VOCs by W_2_ and W_8_ was also reduced when compared to W_0_ in both groups. However, formaldehyde increased by W_2_ and then reduced by W_8_. As with the present study, several previous studies estimated indoor quality after remodeling with EF material [19,20,22]. One study reported that the concentration of TVOCs decreased two weeks after wallpapering, but increased at six and 10 weeks after wallpapering. Furthermore, formaldehyde concentrations decreased at two weeks and kept decreasing until 10 weeks [20]. The change in VOCs and formaldehyde observed were different from the results of our study.

In contrast, the results of the study by Yoo et al. are identical to the changes in the concentrations of indoor air pollutants observed in our study [22]. Yoo et al. wallpapered with PVC materials and eco-friendly materials in an unoccupied, newly built apartment and measured the concentrations of VOCs and formaldehyde emissions for three months. The results showed that the concentration of TVOCs was higher in the initial phase, followed by a gradual decline as time progressed. For the house built with the PVC materials, compared to those with the eco-friendly material (14 days after eco-friendly wallpapering), it took 50 days more for TVOCs concentration to fall below 1000 μg/m^3^. The changes in formaldehyde concentration were alike in both groups: the concentration reached its peak after seven days and remained below 60 μg/m^3^ as time progressed. However, according to our findings, it is difficult to say that the natural material-coated wallpaper used is more effective than the PVC wallpapers in terms of indoor air quality. In the second week after wallpapering, the rise of the formaldehyde concentration was higher in the EF-wallpaper group than in the PVC-wallpaper group. In the same week, the concentration of TVOCs decreased in the PVC-wallpaper group more than in the EF-wallpaper group. The concentration of VOCs decreased less with the eco-friendly wallpaper, probably because of NVOC abundance. In general, eco-friendly wallpaper contains less PVC and includes materials that are paper-backed or simply coated with natural material. One study reported that TVOCs and formaldehyde were less likely to be emitted from the paper-backed materials than materials coated with natural substances [14]. This difference between our finding and Yoo’s findings is probably because we used kenaf as the eco-friendly material, whereas Yoo et al. used PP (polypropylene) resin.

Finally, we studied the positive effects of indoor environment improvements on AD symptoms. AD is a chronically relapsing skin disease characterized by dermal inflammatory infiltrate containing eosinophils. To objectively evaluate AD symptom improvement, we used ECP levels and the SCORAD index to examine the effects of improved indoor air quality [26,27]. In our study, the eosinophil count or ECP showed no statistical significance. Interestingly, an improvement in the SCORAD index was observed during the study period. The decrease in the SCORAD index from W_0_ to W_8_ was significantly greater in the EF-wallpaper group than in the PVC-wallpaper group (Table 6). These findings were consistent with those reported in previous studies [19,20]. In both groups, statistically significant improvements in the patients’ symptoms were observed when the TVOCs and formaldehyde contents decreased by the eighth week after wallpapering, but not in the second week. In our air quality evaluation in the second week after wallpapering, the result of the EF-wallpaper group was not significantly different from that of the PVC-wallpaper group, but the EF-wallpaper group showed an improved SCORAD index result. We assumed that the natural-coated wallpaper emits NVOC that positively affects AD symptoms. Therefore, a generalized linear model procedure was used to determine the pollutants associated with the SCORAD index. The SCORAD index showed a positive correlation with TVOCs and 5VOCs, except styrene, and showed a negative correlation with NVOC % (Table 7). This indicated that the symptoms associated with AD were worse when the concentrations of VOCs and formaldehyde were higher. Additionally, the eco-friendly wallpaper, which had a small reduction in the ratio of VOCs concentration after two weeks, showed better improvements in terms of clinical signs than the PVC wallpapers, probably because of the NVOCs that have risen in the same weeks. Most VOCs are generated artificially, but some are derived from natural sources and named NVOCs [16]. NVOCs that are emitted by forests are believed to be beneficial for health in comparison with other VOCs. Phytoncides are the representative material of NVOCs. Chemical and pharmacological studies on phytoncides have reported that they have anti-inflammatory and anti-oxidant activities [28,29]. Furthermore, they are reportedly associated with an amelioration in conditions such as allergies, multiple sclerosis, and Parkinson’s disease [30]. Interestingly, the concentration of NVOCs at two and eight weeks was statistically higher in the EF-wallpaper group than in the PVC-wallpaper group (Table 5). In addition, the results of our study showed that the SCORAD index was negatively correlated with NVOC/VOCs (NVOC %). This is the first study to show that the higher NVOC % values from natural sources may improve AD symptoms.

This study had several limitations. We could not evaluate the persistent effects of wallpapering. However, considering that Yoo et al. [22] reported that the concentrations of VOCs were maintained consistently after 60 days (eight to nine weeks), we assume that the short-term evaluation at eight weeks more accurately reflected the effect of wallpapering. In contrast, long-term observations may be influenced by various factors missed in our study design. Other limitations included the small number of participants. The statistical power of our minimal sample size was 80% with a significance level of 5%. Another limitation is the short-term sampling. Because human activities and environmental conditions before and during sampling have an effect on the concentration of VOCs, short-term sampling can be inappropriate for assessing the average air pollutant concentrations over long periods. However, some effort was put into ensuring consistency in the measurements by following a set pattern of ventilation prior to sampling. In addition, these results do not solely indicate causality with respect to AD symptom improvement and changing wallpaper. Regular visits to the doctor also may have played a role in improving AD symptoms. Thus, adequately applying emollients or topical steroid during the study period may have also elicited improvement in AD symptoms, and thus, these factors cannot be ignored against the setting of EF-wallpaper use. 

Despite these limitations, this is the first pediatric study comparing indoor air pollutant concentrations between EF wallpapering and PVC wallpapering, using the SCORAD index. We also analyzed the effects of air pollutants on AD, particularly the correlation between the concentrations of the pollutants and the symptoms of AD. The results showed the beneficial effects of EF wallpapering on indoor air quality. Furthermore, indoor air quality has an important role in symptom management for children with AD. Further studies are needed to evaluate the long-term effects of EF wallpaper on indoor air quality improvement, with a focus on the effects of NVOCs which are considered to improve AD symptoms.

## 5. Conclusions

This study evaluated the effects of wallpapering on indoor air quality and the symptoms of AD among children. According to our findings, the management of indoor air quality might be helpful for improving AD symptoms. Particularly, NVOCs in the indoor air after EF wallpapering could have a beneficial effect on health.

## Figures and Tables

**Table 1:** Conditions for volatile organic compound (VOC) analysis using a thermal desorption system (TDS).

Items		TDS Conditions
Split Ratio		Splitless
Carrier GAS and TDS flow		He (99.999%), 1 mL/min
Desorption temperature program	Initial temperature	30 °C (holding 3 min)
	Final temperature	60 °C/min, 280 °C, (holding 5 min)
CIS temperature program	Initial temperature	−30 °C (holding 5 min)
	Final temperature	12 °C/min, 280 °C, (holding 5 min)
Transfer line Temperature		300 °C

**Table 2:** Conditions for VOC analysis using GC/MS.

Items		GC/MS Conditions
Split Ratio		10:1
Detector		MSD (5975, Agilent)
Column		HP-VOC 60.0 m × 320 μm × 1.8 μm
Carrier GAS and Column Flow		He (99.999%), 1 mL/min
Temperature program	Initial temperature	50 °C (5 min)
	Heating rate	5 °C/min, 220 °C, (holding 10 min)
	Final temperature	10 °C/min, 250 °C, (holding 5 min)
MS condition	MS source	230 °C
	MS quad	150 °C
	Mode	EI
	Ionization energy	70 eV
	Detection mode	TIC (scan), *m*/*z*: 35~350

**Table 3:** Conditions for formaldehyde analysis using HPLC.

Parameter	Condition
Instrument	HPLC (Hewlett Packard series 1100)
Column	Inno C18 (250 mm × 4.6 mm × 5 µm)
Mobile phase	Acetonitrile/H_2_O = 60/40 (*v*/*v* %)
Detection	UV 360 nm
Flow rate	1.0 mL/min
Injection volume	20 µL

**Table 4:** Clinical characteristics of the study population.

Characteristics	EF Wallpaper Group	PVC Wallpaper Group	*p* ^a^
Number, *n*	15	16	
Gender, male/female, *n*	7/8	5/11	0.38
Age, years	5.34 ± 1.49	5.68 ± 1.79	0.57
Emollient, g	420.80 ± 208.30	375.00 ± 152.40	0.49
Eosinophil count, mm^3^	497.46 ± 447.16	626.31 ± 777.26	0.61
Total IgE, IU/mL	125.12 ± 84.94	191.36 ± 190.73	0.25
ECP ^b^, μg/L	36.17 ± 36.41	59.15 ± 50.62	0.19
SCORAD index			
W_0_^c^	40.20 ± 11.63	35.08 ± 7.29	0.10
Objective SCORAD			
W_0_^c^	28.0 ± 8.0	23.8 ± 3.8	

Data are presented as mean ± standard deviation; *p* < 0.05 is statistically significant; ^a^ Student-*t* test was used to examine differences between the environmentally friendly (EF)-wallpaper and polyvinyl chloride (PVC)-wallpaper groups by gender, age, amount of emollient use, and result of blood samples, and Wilcoxon two-sample tests were used to examine differences between the groups by SCORAD index and objective SCORAD index values. ^b^ ECP: Eosinophilic cationic protein; ^c^ W_0_: Primary hospital visit.

**Table 5:** Indoor air pollutants before and after wallpapering.

Pollutants	Time	EF Wallpaper			PVC Wallpaper			*p*_0_^b^
		Mean	Geo-Mean ^a^	*p*	Mean	Geo-Mean	*p*	
HCHO ^c^ μg/m^3^	W_0_^d^	111.5 ± 34.2	4.7 ± 0.3		85.8 ± 39.5	4.4 ± 0.3		0.03
	W_2_^d^	149.4 ± 54.1	5.0 ± 0.4	0.04	102.1 ± 40.1	4.6 ± 0.4	0.09	0.01
	W_8_^d^	104.7 ± 40.7	4.6 ± 0.4	0.39	90.6 ± 50.6	4.2 ± 1.2	0.53	0.20
TVOC μg/m^3^	W_0_	1095.4 ± 883.7	6.6 ± 1.1		1099.1 ± 1636.6	6.4 ± 1.0		0.57
	W_2_	863.9 ± 1293.3	6.2 ± 0.9	0.34	287.3 ± 148.9	5.5 ± 0.7	0.00	0.02
	W_8_	292.3 ± 187.5	5.5 ± 0.7	0.00	221.6 ± 139.0	5.0 ± 1.5	0.00	0.25
5VOC μg/m^3^	W_0_	234.9 ± 207.0	5.0 ± 1.0		138.1 ± 135.8	4.6 ± 0.8		0.22
	W_2_	87.1 ± 80.4	4.2 ± 0.9	0.00	54.6 ± 36.0	3.7 ± 0.7	0.00	0.17
	W_8_	57.3 ± 53.4	3.7 ± 0.9	0.00	38.4 ± 22.0	3.4 ± 1.0	0.00	0.42
Benzene μg/m^3^	W_0_	11.0 ± 14.0	1.9 ± 1.2		5.5 ± 9.3	1.2 ± 1.0		0.02
	W_2_	2.2 ± 3.6	0.7 ± 1.0	0.00	3.3 ± 4.2	1.0 ± 1.0	0.64	0.14
	W_8_	1.3 ± 2.5	0.5 ± 0.8	0.00	1.1 ± 2.3	0.4 ± 0.8	0.03	0.43
Toluene μg/m^3^	W_0_	150.0 ± 183.0	4.5 ± 1.1		104.0 ± 126.0	4.2 ± 0.9		0.47
	W_2_	45.5 ± 34.3	3.6 ± 0.9	0.00	25.3 ± 28.4	3.3 ± 0.7	0.00	0.46
	W_8_	30.2 ± 23.6	3.1 ± 0.9	0.00	24.8 ± 13.4	3.0 ± 0.9	0.00	0.74
Ethyl-benzene μg/m^3^	W_0_	18.1 ± 14.6	2.7 ± 0.8		12.4 ± 7.6	2.5 ± 0.5		0.40
	W_2_	11.0 ± 15.5	2.1 ± 0.9	0.00	5.4 ± 3.6	1.7 ± 0.7	0.00	0.18
	W_8_	6.2 ± 5.8	1.7 ± 0.7	0.00	4.6 ± 2.8	1.6 ± 0.6	0.00	0.47
Xylene μg/m^3^	W_0_	27.1 ± 32.2	2.8 ± 1.0		13.1 ± 7.3	2.5 ± 0.5		0.30
	W_2_	15.6 ± 30.2	2.1 ± 1.1	0.00	5.20 ± 3.2	1.7 ± 0.6	0.00	0.20
	W_8_	8.36 ± 8.2	1.9 ± 0.9	0.00	4.9 ± 2.8	1.6 ± 0.6	0.00	0.40
Styrene μg/m^3^	W_0_	28.9 ± 60.4	2.1 ± 1.6		3.2 ± 3.4	1.0 ± 1.0		0.02
	W_2_	13.0 ± 32.7	1.5 ± 1.4	0.10	2.4 ± 3.2	0.8 ± 0.9	0.55	0.11
	W_8_	11.2 ± 34.5	0.9 ± 1.5	0.00	3.1 ± 4.0	1.0 ± 0.9	0.97	0.24
NVOC μg/m^3^	W_0_	314.8 ± 675.8	4.8 ± 1.3		94.5 ± 69.8	4.3 ± 0.8		0.19
	W_2_	448.6 ± 1280.2	4.4 ± 1.6	0.45	34.3 ± 25.6	3.4 ± 0.6	0.00	0.03
	W_8_	66.1 ± 61.8	3.8 ± 0.9	0.00	41.1 ± 46.6	3.2 ± 1.2	0.00	0.14
NVOC % ^c^	W_0_	21.7 ± 18.4			12.3 ± 9.5			0.19
	W_2_	25.3 ± 26.1		0.57	17.5 ± 21.2		0.61	0.20
	W_8_	23.9 ± 17.4		0.51	20.3 ± 17.1		0.09	0.28
TSB ^c^ CFU/m^3^	W_0_	1410.5 ± 772.2	7.1 ± 0.5		1363.0 ± 478.1	7.2 ± 0.3		0.75
	W_2_	2967.3 ± 2309.2	7.8 ± 0.7	0.00	2609.7 ± 1797.2	7.6 ± 0.7	0.00	0.63
	W_8_	1849.0 ± 1345.1	7.3 ± 0.7	0.37	1806.3 ± 1122.5	6.9 ± 2.0	0.62	0.47

Data are presented as mean ± standard deviation; *p* < 0.05 was considered statistically significant as calculated by a paired *t*-test between W_0_ and W_2_ in each group, and also between W_0_ and W_8_; ^a^ Geo-Mean: Indoor air pollutants were transformed to a logarithmic scale because they followed a log-normal distribution, except NVOC%; ^b^ *p*_0_: Comparison between the EF-wallpaper and PVC-wallpaper group as calculated by Wilcoxon two-sample tests; ^c^ HCHO: Formaldehyde, TVOC: Total VOCs; 5VOC: benzene, toluene, ethylbenzene, xylene, styrene, and 2-ethyl-1-hexanol (2E1H) levels; NVOC: Natural VOC; TSB: Total Suspended Bacteria, NVOC % = (NVOC ÷ TVOCs) × 100; CFU: Colony forming unit; ^d^ W_0_: Before wallpapering; W_2_: Two weeks after wallpapering; W_8_: Eight weeks after wallpapering.

**Table 6:** Eosinophil count, eosinophil cationic protein concentration, and SCORAD Index before and after wallpapering.

Variable	Measuring Time	EF Wallpaper		PVC Wallpaper		*p*_0_^a^
		Mean	*p*	Mean	*p*	
ECP ng/mL	W_0_^b^	34.1 ± 35.8		53.7 ± 49.5		
	W_2_^b^	28.6 ± 21.2	0.65	69.8 ± 63.7	0.35	
	W_8_^b^	21.7 ± 15.6	0.13	335 ± 20.4	0.19	
Eosinophil count/mm^3^	W_0_	463.1 ± 423.8		616.7 ± 721.5		
	W_2_	361.1 ± 217.6	0.27	604.2 ± 310.4	0.99	
	W_8_	385.5 ± 234.1	0.48	584.7 ± 319.7	0.80	
SCORAD index	W_0_	40.2 ± 0.2		35.1 ± 5.1		0.10
	W_2_	33.9 ± 3.9	0.02	33.0 ± 3.0	0.41	0.48
	W_8_	23.7 ± 3.7	0.00	23.2 ± 3.2	0.00	0.46
	W_2_–W_0_^c^	−6.20 ± 9.04		−2.04 ± 9.81		0.14
	W_8_–W_0_^c^	−16.46 ± 13.00		−11.49 ± 8.04		0.05 ^‡^
Objective SCORAD index	W_0_	28.0 ± 8.0		23.8 ± 3.8		
	W_2_	24.4 ± 4.4	0.02	22.4 ± 2.4	0.40	
	W_8_	11.7 ± 1.7	0.00	16.0 ± 6.0	0.00	
	W_2_–W_0_	−3.70 ± 5.21		−1.44 ± 6.44		0.10
	W_8_–W_0_	−11.20 ± 9.89		−7.80 ± 5.03		0.08

Data are presented as mean ± standard deviation; *p* < 0.05 was considered statistically significant as calculated by a paired *t*-test between W_0_ and W_2_ in each group, and also between W_0_ and W_8_; ^a^ *p*_0_: Comparison between the EF-wallpaper and PVC-wallpaper groups as calculated by Wilcoxon two-sample tests; ^b^ W_0_: Before wallpapering; W_2_: Two weeks after wallpapering; W_8_: Eight weeks after wallpapering; ^c^ W_2_–W_0_: Comparison of before and two weeks after wallpapering, W_8_–W_0_: Comparison of before and eight weeks after wallpapering. ^‡^ The decrease in the SCORAD was greater in the EF-wallpaper group than in the PVC-wallpaper group though of no statistical significance.

**Table 7:** Correlation between indoor air pollutants and the SCORAD index.

Indoor Pollutants	Adjusted ^a^			Unadjusted		
	β ^b^	STD Error	*p*	β	STD Error	*p*
Formaldehyde	5.95	±2.50	0.02	4.73	±2.61	0.08
Benzene	3.85	±0.93	0.00	4.02	±0.90	0.00
Toluene	6.14	±1.07	0.00	6.33	±1.02	0.00
Ethylbenzene	5.75	±1.35	0.00	5.88	±1.30	0.00
Xylene	5.75	±1.26	0.00	5.49	±1.25	0.00
Styrene	1.06	±0.92	0.25	0.76	±0.86	0.38
TVOCs	3.41	±1.10	0.00	3.49	±1.10	0.00
5VOCs	6.35	±1.09	0.00	6.66	±1.05	0.00
NVOCs	1.89	±0.99	0.06	1.5	±0.98	0.13
NVOC %	−0.13	±0.06	0.02	−0.14	±0.06	0.01
TBS	−2.06	±1.79	0.2541	−1.13	±1.73	0.51

Adjusted ^a^: Adjusted by gender, age, and total amount of emollient use; β ^b^: SCORAD index/ln(μg/m^3^).

## Abbreviations

The following abbreviations are used in this manuscript:

AD	Atopic dermatitis
VOCs	Volatile Organic Compounds
SCORAD	Scoring Atopic Dermatitis
IRB	Institutional Review Board
PVC	Polyvinyl Chloride
TVOCs	Total Volatile Organic Compounds
NVOCs	Natural Volatile Organic Compounds
ECP	Eosinophil Cationic Protein
